# Perioperative textbook outcomes of minimally invasive pancreatoduodenectomy: a multicenter retrospective cohort analysis in a Korean minimally invasive pancreatic surgery registry

**DOI:** 10.1097/JS9.0000000000001390

**Published:** 2024-04-03

**Authors:** Jaewoo Kwon, Chang Moo Kang, Jin-Young Jang, Yoo-Seok Yoon, Hyung Jun Kwon, In Seok Choi, Hee Joon Kim, Sang Hyun Shin, Sang Hyun Kang, Hyung Hwan Moon, Dae Wook Hwang, Song Cheol Kim

**Affiliations:** a Department of Surgery, Kangbuk Samsung Hospital, Sungkyunkwan University School of Medicine; b Department of Hepatobiliary and Pancreatic Surgery, Yonsei University College of Medicine; c Department of Surgery and Cancer Research Institute, Seoul National University College of Medicine; d Division of Hepatobiliary–Pancreatic Surgery, Department of Surgery, Samsung Medical Center, Sungkyunkwan University School of Medicine; e Division of Hepato-Biliary and Pancreatic Surgery, Department of Surgery, University of Ulsan College of Medicine and Asan Medical Center, Seoul; f Department of Surgery, Konyang University Hospital, Konyang University College of Medicine, Daejeon; g Division of Hepato-Pancreato-Biliary Surgery, Department of Surgery, Chonnam National University Medical School, Gwangju; h Department of Surgery, Seoul National University Bundang Hospital, Seoul National University College of Medicine, Seongnam; i Department of Surgery, College of Medicine, Inje University, Busan Paik Hospital; j Department of Surgery, College of Medicine at Kosin University, Busan; k Department of Surgery, Kyungpook National University Medical Center, Daegu, Republic of Korea

**Keywords:** minimally invasive pancreatoduodenectomy, textbook outcome of pancreatic surgery

## Abstract

**Background:**

The aim of this study is to investigate the perioperative composite textbook outcomes of pancreatic surgery after minimally invasive pancreatoduodenectomy (MIPD).

**Materials and methods:**

The cohort study was conducted across 10 institutions and included 1552 patients who underwent MIPD registered with the Korean Study Group on Minimally Invasive Pancreatic Surgery between May 2007 and April 2020. We analyzed perioperative textbook outcomes of pancreatic surgery after MIPD. Subgroup analyses were performed to assess outcomes based on the hospital volume of MIPD.

**Results:**

Among all patients, 21.8% underwent robotic pancreatoduodenectomy. High-volume centers (performing >20 MIPD/year) performed 88.1% of the procedures. The incidence of clinically relevant postoperative pancreatic fistula was 11.5%. Severe complications (Clavien–Dindo grade ≥IIIa) occurred in 15.1% of the cases. The 90-day mortality rate was 0.8%. The mean hospital stay was 13.7 days. Textbook outcomes of pancreatic surgery success were achieved in 60.4% of patients, with higher rates observed in high-volume centers than in low-volume centers (62.2% vs. 44.7%, *P*<0.001). High-volume centers exhibited significantly lower conversion rates (5.4% vs. 12.5%, *P*=0.001), lower 90-day mortality (0.5% vs. 2.7%, *P*=0.001), and lower 90-day readmission rates (4.5% vs. 9.6%, *P*=0.006) than those low-volume centers.

**Conclusion:**

MIPD could be performed safely with permissible perioperative outcomes, including textbook outcomes of pancreatic surgery, particularly in experienced centers. The findings of this study provided valuable insights for guiding surgical treatment decisions in periampullary disease.

## Introductions

Highlights

- This study analyzed 1552 patients who underwent minimally invasive pancreatoduodenectomy (MIPD) at 10 institutions and were registered with the Korean Study Group on Minimally Invasive Pancreatic Surgery.
- The results indicated acceptable perioperative outcomes, including 11.5% for postoperative pancreatic fistulas, 15.1% for severe complications, 0.8% for 90-day mortality, and 60.4% for successful textbook outcomes of pancreatic surgery.
- With the increasing prevalence of MIPD, the results of this study provide valuable insights for guiding surgical treatment decisions for periampullary disease.

Pancreatoduodenectomy (PD) is a highly complicated abdominal surgical procedure, which procedure becomes considerably more challenging when performed using minimally invasive techniques. The first laparoscopic PD (LPD) was reported by Gagner in 1994^1^, and robotic PD (RPD) was first reported by Giulianotti *et al*. in 2003^2^. Since these first minimally invasive PDs (MIPDs) were introduced, various institutions have adopted these techniques. Furthermore, the effectiveness of MIPD has been examined in several studies, including randomized studies, yielding mixed results^3–6^. A meta-analysis of randomized controlled trials comparing LPD with open PD (OPD) also concluded that LPD had no advantage over OPD. However, this analysis noted a high risk of bias and moderate-to-low level of evidence^7^. Although it is still early to make generalized conclusions regarding MIPD, primarily due to the selection bias inherent in retrospective studies and the limited number of prospective studies, the frequency of MIPD is gradually increasing^8–10^. Studies on the learning curve and training for MIPD have emphasized the need for an MIPD registry to gather evidence for clinical research^11–13^. Korean groups have published studies on MIPD and collaborated to establish a registry in line with global trends^9,14–18^. After the establishment of the Korean study group on Minimally Invasive Pancreatic Surgery (K-MIPS) in 2019 (http://kmips.or.kr/index.html), patients who underwent MIPS were retrospectively and prospectively registered.

Textbook outcomes after pancreatic surgery (TOPS) have recently emerged as a valuable approach for assessing surgical success. TOPS considers multiple quality measures representing the standard or benchmark outcomes that surgeons and medical professionals strive to achieve in pancreatic surgery, providing a comprehensive indication of overall outcomes^19–23^.

In this study, we aimed to investigate the perioperative standard TOPS of MIPD using nationwide databases with a large number of cases. In addition, we compared subgroups classified by hospital volume to determine whether hospital volume affects perioperative outcomes.

## Materials and methods

### Study setting and population

This retrospective multicenter cohort study included 1552 patients who underwent MIPD performed by 24 pancreatic surgeons between May 2007 and April 2020 across 10 institutions in Korea. Consecutive patients who underwent intended MIPD at each institution were included. MIPD included both LPD and RPD. RPD included laparoscopic resection with robotic reconstruction or robotic resection and reconstruction. The research was conducted using data registered in the K-MIPS registry. This study was approved by the institutional review board of Asan Medical Center (Approval number: 2020-0600) and of each participating center. Patients’ informed consent was waived because of the retrospective observational nature of this study. Inclusion and exclusion criteria were based on the judgment of the operator in each institution. This study followed the Strengthening The Reporting Of Cohort Studies in Surgery (STROCSS) guidelines^24^ (Supplemental Digital Content 1, http://links.lww.com/JS9/C242).

### Data collection

Clinical data were collected and analyzed. Postoperative complications were classified according to the Clavien–Dindo classification system^25^. Post-pancreatectomy complications, such as postoperative pancreatic fistula (POPF)^26^, delayed gastric emptying(DGE)^27^, postoperative pancreatic hemorrhage(PPH)^28^, and chyle leakage^29^, were defined and graded based on the International Study Group of Pancreatic Surgery definitions. Bile leakage was defined and graded according to the International Study Group of Liver Surgery criteria^30^. TOPS was defined based on the Dutch Pancreatic Cancer Group criteria^23^, which include the absence of POPF, bile leakage, PPH, Clavien–Dindo ≥grade III complications, readmission within 90 days, in-hospital or 90-day mortality, or postoperative hospital stay exceeding 14 days. The classifications of malignancy and benign conditions were based on the 2019 World Health Organization classification for tumors of the digestive system^31^. Although all institutions participating in this study were tertiary referral hospitals, they were stratified to evaluate potential differences in postoperative outcomes and TOPS according to the number of MIPDs performed per year as follows: a high-volume center performing >20 MIPDs/year and a low-volume center performing ≤20 MIPDs/year. Additionally, the analysis was repeated, excluding all patients with missing postoperative outcome values for a more comprehensive assessment.

### Statistical analysis

Statistical analyses involved reporting demographics, along with operative, postoperative, and pathological outcomes. Continuous variables were expressed as means±standard deviations and compared using the Student’s *t*-test, when applicable. Categorical variables were compared using the *χ*^2^, Fisher’s exact, or linear-by-linear association test. Institutions participating in this study were divided into two groups based on the number of MIPDs performed (>20 MIPDs/year vs. ≤20 MIPDs/year). These groups were then compared in terms of demographics, perioperative outcomes, and TOPS. And to calculate the TOPS success rate more accurately, the current study performed another analysis on 1239 patients, excluding patients with missing values for postoperative outcomes. For exploratory analysis, we compared the postoperative outcomes based on the number of MIPDs (>50 MIPDs/year vs. 20–50 MIPDs/year vs. <20 MIPDs/year). To compare the three groups, this study utilized analysis of variance for continuous variables and the *χ*^2^ test for categorical variables. Subsequently, a Bonferroni-corrected post-hoc analysis was performed to identify significant group differences. Demographics and perioperative outcomes were also compared between LPD and RPD. Statistical analyses were conducted using SPSS Statistics for Windows, version 28.0 (IBM Corp., Armonk, NY, USA) and R version 4.2.2 (R Foundation for Statistical Computing). Statistical significance was set at *P*<0.05

## Results

### Study population

Among the 10 institutions participating in the study, 4, 2, and 4 performed >100, 50–100, and <50 PDs per year, respectively. When the number of MIPDs performed at each center was averaged by year, 2, 2, and 6 institutions were observed to have performed >50, 20–50, and <20 MIPDs per year, respectively. Among all patients, 88.1% (*n*=1368) were treated in hospitals that performed >20 MIPDs per year (Supplementary Fig. 1, Supplemental Digital Content 2, http://links.lww.com/JS9/C243). Among the 24 surgeons who participated in this study, 19, 3, and 2 had performed >100, 50–100, and <50 OPDs, respectively, prior to performing MIPD. The mean age of the patients was 61.5±13.0 years and 48.9% of the patients were women (*n*=759). Approximately 84.6% of the patients had malignancies. RPD was performed in 21.8% of the cases, with laparoscopic resection and robotic reconstruction being more frequently performed (*n*=286, 84.4%) than robotic resection and anastomosis (*n*=49, 14.5%). Open conversion rate was 6.3% (*n*=97).

Among the patients, 3.5% underwent concomitant major vascular resection. The duct-to-mucosa technique (72.8%) was the most frequently employed method for pancreaticojejunostomy, followed by the dunking method. The mean estimated blood loss was 297±481 ml, and the mean operation time was 417±103 min. Detailed demographics are summarized in Supplementary Table 1 (Supplemental Digital Content 2, http://links.lww.com/JS9/C243).

### Perioperative and TOPS outcomes

Postoperative complications and pathologic outcomes are presented in Table 1. The mean hospital stay after surgery was 13.7±10.9 days. Clavien–Dindo ≥grade III complications occurred in 15.1% of the cases. Clinically relevant POPF was observed in 11.5% of the patients. Additionally, DGE (5.1%), PPH (4.0%), chyle leakage (5.5%), and bile leakage (2.2%), with a 90-day mortality rate was 0.8%. Other outcomes included complicated fluid collection (17.4%), unplanned intensive unit care (ICU) (2.6%), unplanned reoperation (4.9%), and 90-day readmission (5.0%). The reasons for open conversion are presented in Supplementary Table 2 (Supplemental Digital Content 2, http://links.lww.com/JS9/C243). The most common cause was bleeding (19.6%), followed by adhesion (14.4%), inflammation (12.4%), and difficulty of reconstruction (11.3%). Mean tumor size was observed to be 2.72±1.44 cm, with the most common tumor location being the pancreas (55.1%), followed by ampulla of Vater (23.2%), bile duct (18.3%), and duodenal lesion (3.4%). After excluding 196 cases with missing values, 1356 cases of tumors were evaluated and 1147 (84.6%) were identified as malignant tumors. Among the malignant tumors, 786 (58.0%) were diagnosed as adenocarcinomas, and the mean number of harvested lymph nodes was 13.7±8.0. The R0 resection success rate was 95.5%. After excluding 29 patients with incomplete data, calculations for TOPS success after MIPD were performed on 1523 patients. TOPS success was accomplished in 920 patients (60.4%). The success rate of each module and the cumulative TOPS rate are presented in Table 2. The most common cause of failure was a hospital stay of more than 14 days after surgery (71.5%), followed by Clavien–Dindo grade ≥IIIa (38.8%), the occurrence of POPF (28.5%), 90-day readmission (12.6%), PPH (10.3%), bile leakage (5.6%), and 90-day mortality (2.0%).

**Table 1:** Postoperative complications and pathologic outcomes.

Variables	*N*=1552^a^	Missing, *N* (%)
Hospital stays after operation, days (±SD)		
Mean	13.7 (±10.9)	
Median (IQR)	10 (9–14)	
Complication grade, *n* (%)		
None	719 (46.3)	
Grade I–II	599 (38.6)	
≥Grade IIIa	234 (15.1)	
POPF, *n* (%)		
None or biochemical leakage	1327 (88.5)	53 (3.4)
Grade B	162 (10.8)	
Grade C	10 (0.7)	
Delayed gastric emptying, *n* (%)		
Yes	76 (5.1)	52 (3.4)
Grade A	42 (2.8)	
Grade B	25 (1.7)	
Grade C	9 (0.6)	
No	1424 (94.9)	
Post-pancreatectomy hemorrhage, *n* (%)		
Yes	62 (4.0)	
Grade A	1 (0.1)	
Grade B	18 (1.2)	
Grade C	29 (1.8)	
Unknown	14 (0.9)	
No	1490 (96.0)	
Chyle leakage, *n* (%)		
Yes	82 (5.5)	55 (3.5)
Grade A	81 (5.4)	
Grade B	1 (0.1)	
Grade C	0 (0.0)	
No	1415 (94.5)	
Bile leakage, *n* (%)		
Yes	34 (2.2)	
Grade A	3 (0.2)	
Grade B	13 (0.8)	
Grade C	3 (0.2)	
Unknown	15 (1.0)	
No	1518 (97.8)	
Complicated fluid collection, *n* (%)		
Yes	217 (17.4)	303 (19.5)
No	1032 (82.6)	
Unplanned ICU care, *n* (%)		
Yes	32 (2.6)	303 (19.5)
No	1217 (97.4)	
Unplanned reoperation, *n* (%)		
Yes	61 (4.9)	303 (19.5)
No	1188 (95.1)	
90-day mortality, *n* (%)		
Yes	12 (0.8)	1 (<0.1)
No	1539 (99.2)	
90-day readmission, *n* (%)		
Yes	76 (5.0)	29 (1.9)
No	1447 (95.0)	
Textbook outcome of pancreatic surgery, *n* (%)		
Yes	920 (60.4)	29 (1.9)
No	603 (39.6)	
Pathologic tumor size, cm (±SD)		
Mean	2.72 (±1.44)	
Tumor location, *n* (%)		
Pancreas	853 (55.1)	
Ampulla of Vater	359 (23.2)	
Bile duct	284 (18.3)	
Duodenum	53 (3.4)	
Malignancy, *n* (%)		
Malignancy	1147 (84.6)	196 (12.6)
Adenocarcinoma	786 (58.0)	
Other carcinoma	40 (2.9)	
NET	143 (10.5)	
IPMN	64 (4.7)	
SPN	82 (6.0)	
GIST	30 (2.2)	
No residual tumor	2 (0.1)	
Benign	209 (15.4%)	
Number of harvested lymph nodes, *n* (±SD)		
Mean	13.7 (±8.0)	
Number of positive lymph nodes, *n* (±SD)		
Mean	0.7 (±1.8)	
Resection margin^b^, *n* (%)		
R0	1088 (94.8)	2 (0.2)
R1	55 (5.1)	
R2	2 (0.2)	

a Data are presented as the number (percentage) of patients unless otherwise indicated.

b Resection margin included only for malignant tumor.

GIST, gastrointestinal stromal tumor; ICU, intensive care unit; IPMN, intraductal papillary mucinous neoplasm; NET, neuroendocrine tumor; POPF, postoperative pancreatic fistula; SD, standard deviation; SPN, solid pseudopapillary tumor.

**Table 2:** Textbook outcome of pancreatic surgery assessed individually and cumulatively.

Variables	*N*=1552	Missing, *N* (%)
90-day mortality, *n* (%)		
Yes	12 (0.8)	1 (0.1)
No	1539 (99.2)	
POPF, *n* (%)		
Grade B or C	172 (11.5)	53 (3.4)
None or biochemical leakage	1327 (88.5)	
Post-pancreatectomy hemorrhage, *n* (%)		
Yes	62 (4.0)	
No	1490 (96.0)	
Bile leakage, *n* (%)		
Yes	34 (2.2)	
No	1518 (97.8)	
Complications grade^a^, *n* (%)		
≥Grade IIIa	234 (15.1)	
No or ≤Grade II	1318 (84.9)	
90-day readmission, *n* (%)		
Yes	76 (5.0)	29 (1.9)
No	1447 (95.0)	
Discharge after 14 days of surgery		
Yes	431 (28.2)	25 (1.6)
No	1096 (71.8)	
Cumulative textbook outcome of pancreatic surgery, *n* (%)		
Success	920 (60.4)	29 (1.9)
Fail	603 (39.6)	

POPF, postoperative pancreatic fistula.

a Complication grade was classified according to the Clavien-Dindo classification(25).

### Comparative analysis of outcomes based on the hospital volume of MIPD

**Figure 1:**
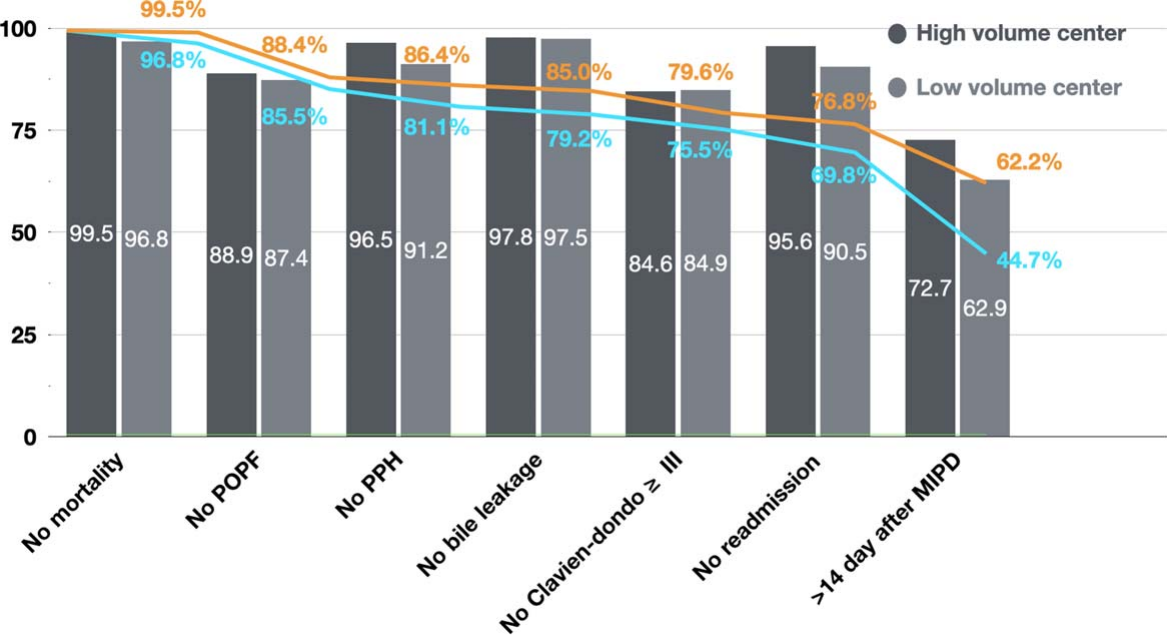
Textbook outcome of pancreatic surgery (TOPS) categorized based on hospital volume. The textbook outcome of pancreatic surgery is assessed individually and cumulatively. Each factor’s percentage was measured, excluding missing values. The orange line (high-volume center) and blue line (low-volume center) indicate the TOPS success rate, excluding cases that do not meet each TOPS item. The TOPS success rate was higher in high-volume centers than in low-volume centers (62.2% vs. 44.7%, *P*<0.001). Interestingly there was no significant difference according to hospital volume except for hospital stays over 14 days after operation (76.8% vs. 69.8%, *P*=0.052). MIPS, minimally invasive pancreatoduodenectomy; POPF, postoperative pancreatic fistula; PPH, postoperative pancreatic hemorrhage.

Demographics and perioperative outcomes based on hospital volume (high volume vs. low volume) of MIPD cases are presented in Table 3. Compared to patients from low-volume centers, those who underwent MIPD at high-volume centers were younger (61.0±13.2 years vs. 65.3±11.2 years, *P*<0.001), with a higher proportion of female patients (50.4% vs. 38.0%, *P*=0.002) Additionally, patients from high-volume centers had lower ASA (American Society of Anesthesiology) scores (*P*=0.007) than those from low-volume centers. In low-volume centers, there were more cases where MIPD was performed recently compared to high-volume centers (*P*<0.001). The proportion of RPD was higher in high-volume centers (24.2% vs. 4.3%, *P*<0.001), whereas the open conversion rate was higher in low-volume centers (5.4% vs. 12.5%, *P*=0.001). Transfusion rate (9.1% vs. 20.1%, *P*<0.001) and the operation time (407±97 min vs. 483±117 min, *P*<0.001) were also higher in patients from low-volume centers. The proportion of PPH (3.5% vs. 7.6%, *P*=0.008), unplanned ICU care (1.8% vs. 8.9%, *P*<0.001), 90-day mortality (0.5% vs. 2.7%, *P*<0.001), and 90-day readmission (4.5% vs. 9.6%, *P*=0.006) were also higher in low-volume centers. However, estimated blood loss, hospital stay after operation, rate of Clavien–Dindo grade ≥III complications, clinically relevant POPF, DGE, bile leakage, and unplanned reoperation did not significantly differ with volume. The TOPS success rate was higher in high-volume centers than in low-volume centers (62.2% vs. 44.7%, *P*<0.001) (Fig. 1). Interestingly, when the TOPS success rate was calculated using six factors, excluding postoperative hospital stay, there was no difference between the two groups (76.8% vs. 69.8%, *P*=0.052).

**Table 3:** Demographics and perioperative outcomes according to MIPD cases.

Variables	High volume (>20 MIPDs/year, *n*=1368)	Low volume (≤20 MIPDs/year, *n*=184)	*P*
Age, years (±SD)			
Mean	61.0 (±13.2)	65.3 (±11.2)	<0.001^a^
Median (IQR)	62 (54–71)	66 (59–74)	
Sex, *n* (%)			
Female	689 (50.4)	70 (38.0)	0.002^b^
Male	679 (49.6)	114 (62.0)	
BMI, kg/m^2^ (±SD)			
Mean	23.1 (±2.7)	23.1 (±2.9)	0.923^a^
Median (IQR)	23.0 (21.3–24.9)	22.8 (21.2–24.8)	
ASA score, *n* (%)			
I	206 (15.1)	25 (13.6)	0.007^b^
II	985 (72.0)	150 (81.5)	
III	174 (12.7)	9 (4.9)	
IV	3 (0.2)	0 (0.0)	
Operation year, *n* (%)			
≤2016	550 (40.2)	25 (13.6)	<0.001^b^
2017	278 (20.3)	28 (15.2)	
2018	229 (16.7)	41 (22.3)	
≥2019	311 (22.7)	90 (48.9)	
Type of minimally invasive surgery, *n* (%)			
Laparoscopic	963 (70.4)	153 (83.2)	<0.001^b^
Robotic	331 (24.2)	8 (4.3)	
Open conversion	74 (5.4)	23 (12.5)	
Operation method, *n* (%)			
PPPD	1210 (88.5)	163 (92.6)	<0.001^b^
PrPD	98 (7.2)	4 (2.3)	
Other	60 (4.4)	9 (5.1)	
NA	0	8	
Additional organ resection, *n* (%)			
Yes	27 (2.0)	5 (2.7)	0.576^b^
No	1341 (98.0)	179 (97.3)	
Additional major vessel resection, *n* (%)			
Yes	54 (3.9)	1 (0.5)	0.017^b^
No	1314 (96.1)	183 (99.5)	
Transfusion, *n* (%)			
Yes	124 (9.1)	37 (20.1)	<0.001^b^
No	1244 (90.9)	147 (79.9)	
Estimated blood loss, ml (±SD)			
Mean	292 (±493)	352 (±310)	0.238^a^
Median (IQR)	150 (100–300)	250 (150–500)	
Operation time, min (±SD)			
Mean	407 (±97)	483 (±117)	<0.001^a^
Median (IQR)	397 (340–460)	472 (405–530)	
Hospital stay after operation, days (±SD)			
Mean	13.5 (±11.0)	15.2 (±10.2)	0.062^a^
Median (IQR)	10 (8–12)	12 (10–15)	
Complications grade^c^, *n* (%)			
None or Grade I or II	1158 (84.6)	160 (87.0)	0.412^b^
≥Grade IIIa	210 (15.4)	24 (13.0)	
POPF, *n* (%)			
None or biochemical leakage	1212 (88.9)	115 (85.2)	0.202^b^
Grade B or C	152 (11.1)	20 (14.8)	
NA	4	49	
Delayed gastric emptying, *n* (%)			
Yes	69 (5.1)	7 (5.2)	0.948^b^
No	1296 (94.9)	128 (94.8)	
NA	3	49	
Post-pancreatectomy hemorrhage, *n* (%)			
Yes	48 (3.5)	14 (7.6)	0.008^b^
No	1320 (96.5)	170 (92.4)	
Chyle leakage, *n* (%)			
Yes	81 (5.9)	1 (0.5)	0.005^b^
No	1285 (93.6)	134 (72.8)	
NA	6	49	
Bile leakage, *n* (%)			
Yes	30 (2.2)	4 (2.2)	>0.999^b^
No	1338 (97.8)	180 (97.8)	
Unplanned ICU care, *n* (%)			
Yes	20 (1.8)	12 (8.9)	<0.001^b^
No	1094 (98.2)	123 (91.1)	
NA	254	49	
Unplanned reoperation, *n* (%)			
Yes	52 (4.6)	9 (5.8)	0.292^b^
No	1062 (95.4)	126 (94.2)	
NA	254	49	
90-day mortality, *n* (%)			
Yes	7 (0.5)	5 (2.7)	0.001^b^
No	1361 (99.5)	178 (97.3)	
NA	0	1	
90-day readmission, *n* (%)			
Yes	61 (4.5)	15 (9.6)	0.006^b^
No	1305 (95.5)	142 (90.4)	
NA	2	27	
Textbook outcome of pancreatic surgery, *n* (%)			
Yes	849 (62.2)	71 (44.7)	<0.001^b^
No	515 (37.8)	88 (55.3)	
NA	4	25	

a *P* value=analysis of variance.

b *P* value=Pearson *χ*^2^ test.

c Complication grade was classified according to the Clavien-Dindo classification(25).

ASA score, American Society of Anesthesiology score; BMI, body mass index; ICU, intensive care unit; POPF, postoperative pancreatic fistula; PPPD, pylorus preserving pancreatoduodenectomy; PrPD, pylorus resection pancreatoduodenectomy; SD, standard deviation.

After excluding 313 patients with missing values for at least one postoperative outcome, the perioperative outcomes of both high-volume and low-volume centers were re-evaluated (Supplementary Table 3, Supplemental Digital Content 2, http://links.lww.com/JS9/C243 and Supplementary Fig. 2, Supplemental Digital Content 2, http://links.lww.com/JS9/C243). After excluding missing values, the TOPS success rate increased to 65.5%. In the additional analysis, ASA score was not different between the two groups, but unplanned ICU care (1.8% vs. 8.7%, *P*<0.001), 90-day mortality (0.5% vs. 3.2%, *P*=0.013), and TOPS success rates (66.9% vs. 53.2%, *P*=0.002) were also better in the high-volume center (66.9% vs. 53.2%, *P*=0.002). However, unlike the forward analysis result, there was still a difference in the incidence of TOPS according to hospital volume, except for hospital stays over 14 days after operation (76.6% vs. 68.3%, *P*=0.037). For the exploratory analysis, we compared the postoperative outcomes based on the number of MIPDs (>50 MIPDs/year vs. 20–50 MIPDs/year vs. <20 MIPDs/year) (Supplementary Table 4, Supplemental Digital Content 2, http://links.lww.com/JS9/C243).

### Comparative analysis of outcomes based on the operation method

Table 4 shows demographics and perioperative outcomes between LPD and RPD. Age and sex were not different between the two groups, but BMI was more higher in RPD groups (23.0±2.8 kg/m^2^ vs. 23.5±2.6 kg/m^2^, *P*=0.005). ASA score was better in the RPD group (*P*=0.001). The open conversion rate was higher in the LPD group (7.9% vs. 0.3%, *P*<0.001). Estimated blood loss was higher in the RPD group (262±483 vs. 394±464, *P*<0.001), but the transfusion rate was not different between the two groups (11.1% vs. 7.6%, *P*=0.062). Operation time (428 min vs. 377 min, *P*<0.001) and postoperative hospital stay (14.2 days vs. 11.9 days, *P*=0.001) were shorter in the RPD group. Complication grade, POPF rate, and unplanned ICU care were not different statistically, but DGE (5.8% vs. 2.7%, *P*=0.021), PPH (4.6% vs. 1.8% *P*=0.017), chyle leakage (6.6% vs. 1.8%, *P*=0.001), unplanned reoperation (6.2% vs. 1.0%, *P*<0.001), 90-day readmission rate (6.0% vs. 1.5%, *P*<0.001) were better in RPD group. The success rate of TOPS was also better in the RPD group (57.8% vs. 69.5%, *P*<0.001).

**Table 4:** Demographics and perioperative outcomes according to operation method.

Variables	LPD (*n*=1212)	RPD (*n*=340)	*P*
Age, years (±SD)			
Mean	61.5 (±13.2)	61.4 (±12.4)	0.860^a^
Median (IQR)	63 (54–71)	62 (55–70)	
Sex, *n* (%)			
Female	601 (49.6)	158 (46.5)	0.310^b^
Male	611 (50.4)	182 (53.5)	
BMI, kg/m^2^ (±SD)			
Mean	23.0 (±2.8)	23.5 (±2.6)	0.005^a^
Median (IQR)	22.9 (21.1–24.7)	23.2 (21.7–25.2)	
ASA score, *n* (%)			
I	163 (13.4)	68 (20.0)	0.001^b^
II	889 (73.3)	246 (72.4)	
III	158 (13.0)	25 (7.4)	
IV	2 (0.2)	1 (0.3)	
Hospital volume, *n* (%)			
High	1036 (85.5)	332 (97.6)	<0.001^b^
Low	176 (14.5)	8 (2.4)	
Operation method, *n* (%)			
PPPD	1090 (90.5)	283 (83.2)	<0.001^b^
PrPD	54 (4.5)	48 (14.1)	
Other	60 (5.0)	9 (2.6)	
NA	8	0	
Conversion to open, *n* (%)			
Yes	96 (7.9)	1 (0.3)	<0.001^b^
Additional organ resection, *n* (%)			
Yes	25 (2.1)	7 (2.1)	>0.999^b^
No	1187 (97.9)	333 (97.9)	
Additional major vessel resection, *n* (%)			
Yes	53 (4.4)	2 (0.6)	<0.001^b^
No	1159 (95.6)	338 (99.4)	
Transfusion, *n* (%)			
Yes	135 (11.1)	26 (7.6)	0.062^b^
No	1077 (88.9)	314 (92.4)	
Estimated blood loss, ml (±SD)			
Mean	262 (±483)	394 (±464)	<0.001^a^
Median (IQR)	140 (100–300)	250 (100–500)	
Operation time, min (±SD)			
Mean	428 (±99)	377 (±106)	<0.001^a^
Median (IQR)	419 (360–483)	360 (305–429)	
Hospital stay after operation, days (±SD)			
Mean	14.2 (±11.5)	11.9 (±8.5)	0.001^a^
Median (IQR)	11 (9–15)	9 (8–12)	
Complications grade^c^, *n* (%)			
None	551 (45.5)	168 (49.4)	0.294^b^
Grade I–II	480 (39.6)	119 (35.0)	
≥ Grade IIIa	181 (14.9)	53 (15.6)	
POPF, *n* (%)			
None or biochemical leakage	1022 (88.0)	305 (90.2)	0.262^b^
Grade B or C	139 (12.0)	33 (9.8)	
NA	51	2	
Delayed gastric emptying, *n* (%)			
Yes	67 (5.8)	9 (2.7)	0.021^b^
No	1094 (94.2)	330 (97.3)	
NA	51	1	
Post-pancreatectomy hemorrhage, *n* (%)			
Yes	56 (4.6)	6 (1.8)	0.017^b^
No	1156 (95.4)	334 (98.2)	
Chyle leakage, *n* (%)			
Yes	76 (6.6)	6 (1.8)	0.001^b^
No	1082 (93.4)	333 (98.2)	
NA	54	1	
Bile leakage, *n* (%)			
Yes	24 (2.0)	10 (2.9)	0.285^b^
No	1188 (98.0)	330 (97.1)	
Unplanned ICU care, *n* (%)			
Yes	28 (3.0)	4 (1.3)	0.145^b^
No	910 (97.0)	307 (98.7)	
NA	274	29	
Unplanned reoperation, *n* (%)			
Yes	58 (6.2)	3 (1.0)	<0.001^b^
No	880 (93.8)	308 (99.0)	
NA	274	29	
90-day mortality, *n* (%)			
Yes	8 (0.7)	4 (1.2)	0.460^b^
No	1203 (99.3)	336 (98.8)	
NA	1	0	
90-day readmission, *n* (%)			
Yes	71 (6.0)	5 (1.5)	<0.001^b^
No	1113 (94.0)	334 (98.5)	
NA	28	1	
Textbook outcome of pancreatic surgery, *n* (%)			
Yes	685 (57.8)	235 (69.5)	<0.001^b^
No	500 (42.2)	103 (30.5)	
NA	27	2	

a *P* value=analysis of variance.

b *P* value=Pearson *χ*^2^ test.

c Complication grade was classified according to the Clavien-Dindo classification(25).

ASA score, American Society of Anesthesiology score; BMI, body mass index; ICU, intensive care unit; NA, not available; POPF, postoperative pancreatic fistula; PPPD, pylorus preserving pancreatoduodenectomy; PrPD, pylorus resection pancreatoduodenectomy; SD, standard deviation.

## Discussion

The present study represents the first comprehensive analysis of multi-institutional MIPD data from the nationwide registry in Korea, focusing on the TOPS perioperative performance rate. Notably, this study did not collect data from all institutions that have implemented MIPD in Korea; therefore, experiences are likely to vary among institutions. However, the study included both the most representative institutions with experienced MIPD surgeons and referral institutions with less experienced MIPD surgeons. This makes the findings of this study more informative for patients undergoing MIPD.

The present study revealed that perioperative outcomes in MIPD were comparable to those of other studies, with some factors showing more favorable results^32–36^.

In addition to conventional indicators of surgery, we investigated TOPS, which provides a multidimensional measure for assessing the quality of surgical outcomes as a comprehensive indication of overall outcomes. Previous studies have investigated textbook outcomes after hepatobiliary and pancreatic surgery^19–23^. Heidsma *et al*.^33^ reported that TOPS not only signifies successful surgical outcomes but also offers potential oncological benefits. Based on an analysis of 2633 patients, van Roessel *et al*.^23^ reported a TOPS success rate of 47.5% after PD, including a postoperative hospital stay of >14 days. Lof *et al*. reported a TOPS rate of 56.4% in a cohort of 250 patients who underwent enhanced recovery after PD. They defined prolonged hospital stay as the 25th highest percentile of the total cohort^26^. The US Neuroendocrine Tumor Study Group reported a TOPS rate of 32.0% after PD^33^. Recently, a multicenter retrospective cohort study in China reported a TOPS rate of 56% after LPD^34^. In the present study, we observed a TOPS success rate of 60.4% after MIPD.

All the institutions participating in the current study are tertiary referral institutions with 800 beds or more, but the number of MIPDs performed differed among hospitals. One low-volume center performed fewer than five MIPD procedures annually, whereas the high-volume institution that was the first in South Korea to perform MIPD performed more than 100 MIPD procedures annually. The criteria for distinguishing between high-volume and low-volume centers vary among published studies^8,37,38^; in the current study, the criterion for distinguishing between high and low volume was defined as 20 MIPD per year according to the Miami International Evidence-Based Guideline^13^. When comparing perioperative outcomes based on the annual MIPD volume, several differences were observed between the two groups. First, the demographics differed between the two groups. This difference is likely due to the differences in the locations of the centers. All four high-volume centers are located in metropolitan areas, while only one of the low-volume institutions has a metropolitan location, and the other five are located in rural areas. There are differences in the demographic structures of metropolitan versus rural regions in South Korea; for example, the proportion of older individuals is relatively higher in rural areas than in metropolitan areas^39^. The older the patient, the greater the underlying disease and the higher the operative risk. Due to the demographic differences between these patient groups, careful interpretation of the results is needed. Furthermore, regarding complicated fluid collection, ICU care, and the requirement for reoperation, there were too many missing values for the statistical results to be robust. Further multivariate analyses are needed to identify any significant differences in postoperative outcomes according to institutional volume.

High-volume centers were observed to be superior to low-volume centers in terms of several perioperative outcomes, including transfusion, operation time, PPH, unplanned ICU care, 90-day readmission rate, 90-day readmission rate, and TOPS success rate. Differences in outcomes based on hospital volume have been published in several studies. Adam *et al*.^35^ reported that the MIPD hospital volume is significantly associated with improved MIPD outcomes, with a threshold of 22 cases per year. The Miami International Guidelines on minimally invasive pancreas resection reported that center volume strongly affects outcomes after minimally invasive pancreas resection. They emphasized that MIPD should be performed in high-volume centers due to reduced mortality and morbidity^13^. The differences in surgical case experience among surgeons, depending on the hospital volume, can explain this disparity. In our study, we also observed variations in the years of experience in performing MIPD based on hospital volume, indicating potential differences among individual surgeons working in those hospitals. A multicenter retrospective study identified that operator experience influenced complication and mortality rates after MIPD^36^. From this standpoint, including the current study, a cautious recommendation can be made to enhance the TOPS success rate by opting for surgery at an institution that conducts more than 20 MIPD annually.

Similar to previous reports, the current study confirmed the existence of variations in TOPS scores based on hospital volume. The primary factor contributing to this disparity was the duration of postoperative hospital stay. First, differences in hospital scale may result in variances in interventions and medical environments, influencing the provision of supportive care after complications. Second, Korea’s medical environment, characterized by relatively low medical costs, may allow patients to extend their hospital stay. Nevertheless, the other factors related to TOPS, excluding this aspect, did not exhibit statistically significant differences according to hospital volume. Hence, the establishment of a registry system to disseminate countermeasures against complications and the implementation of a specialized complication response system in low-volume centers could enhance the success rate of TOPS.

Recently, a retrospective multicenter analysis conducted in China in 2021 reported no differences in complications other than the length of hospital stay according to hospital volume. Interestingly, they observed differences in length of stay, POPF, operation failure, and 30-day mortality depending on the surgeon’s experience^36^. In the present study, the occurrence of severe complications, classified as Clavien–Dindo grade ≥III (a critical criterion for postoperative lethal outcomes), did not demonstrate statistically significant differences based on the hospital volume of MIPD (15.4% vs. 13.0%, *P*=0.412). Similarly, no statistically significant differences were observed in the clinically relevant POPF rates according to the hospital volume of MIPD. When comparing the success rates using the six TOPS factors excluding the postoperative hospital stay, there was no statistical difference between hospital volumes (76.8% vs. 69.8%, *P*=0.052). In the current study, 19 of the 24 surgeons started MIPD after experiencing at least 100 cases of open OPD. These findings suggested that surgeons with a certain level of PD experience can quickly reduce the risk of TOPS failure associated with MIPD.

In addition to PD experience, the potential dissemination of surgical knowledge and expertise on MIPD through academic society networks and academic collaborations facilitates the sharing of clinical experiences and best practices among surgeons. This, in turn, can reduce outcome disparities based on the hospital volume of MIPD. The K-MIPS convenes quarterly academic meetings to foster discussions on the clinical experiences and future directions of MIPD. Hence, we recommend all institutions conducting MIPDs participate in a prospective registry to monitor the safety and efficacy of these operations. Moreover, we suggest that sharing the registry’s results could facilitate the dissemination of indirect experiences and promote knowledge exchange among healthcare professionals, enabling the adoption of best practices and ultimately improving patient outcomes.

This study additionally compared perioperative outcomes according to surgical methods. Comparative studies of LPD and RPD are currently underway worldwide. A recently published meta-analysis reported that RPD represents less blood loss and fewer wound infections^40^. Kamarajah *et al*.^41^ also reported that RPD was associated with lower open conversion and transfusion rates than LPD. In the current study, RPD also had a lower open conversion rate, shorter operative time, and shorter postoperative hospital stay than LPD. Additionally, it was superior in DGE, PPH, chyle leakage, unplanned reoperation, readmission, and TOPS. However, these results should be interpreted with caution. A total of 84.4% of RPDs in the current study were hybrid operations that underwent laparoscopic resection followed by robotic reconstruction. Second, robotic surgery is still relatively expensive in Korean society; thus, it is targeted at patients with financial means who have private insurance, and selection bias may occur owing to the surgeon’s burden of surgery. Therefore, further research is required to determine the superiority of RPD.

### Strengths and limitations

The present study had several limitations. First, this study did not include a comparison to open surgery due to the retrospective nature of the data. Thus, further research utilizing prospective randomized comparative designs with larger sample sizes is needed to confirm the safety and efficacy of MIPD and guide clinical practice. Second, not all institutions performing MIPD in Korea participated in this study; therefore, caution is necessary when generalizing the study results. Third, the study data was collected retrospectively, and there may have been missing values, necessitating caution in the interpretation of the results. Despite these limitations, this study holds value as it presents the first analysis results from the MIPD registry of the K-MIPS. Furthermore, to the best of our knowledge, this is the first multicenter study to report on the success rate of achieving TOPS after MIPD.

## Conclusions

The multicenter registry of K-MIPS demonstrated that MIPD is currently being performed safely, with acceptable perioperative outcomes, including TOPS, particularly in experienced centers. However, improving postoperative outcomes in low-volume centers remains an important task for the continuous advancement of minimally invasive pancreatic surgery. As the frequency of MIPD continues to gradually increase, the results of this study provide valuable insights for guiding surgical treatment decisions for periampullary disease.

## Ethical approval

This study was approved by the institutional review board of Asan Medical Center (Approval number: 2020-0600) and of each participating center.

## Consent

The patient’s written consent was not required because this study is a retrospective observational cohort study.

## Sources of funding

This study was supported by grants from the Korean Pancreas Surgery Club (grant number: 2022IT0002) and Asan Institute for Life Sciences, Asan Medical Center (grant number: 2020IP0104).

## Author contribution

Song Cheol Kim has full access to all of the data in the study and takes responsibility for the integrity of the data and the accuracy of the data analysis. C.M.K., J.-Y.J., Y.-S.Y., and S.C.K.: conception and design; J.K., C.M.K., J.-Y.J., Y.-S.Y., H.J.K., I.S.C., H.J.K., S.H.S., S.H.K., H.H.M., D.W.H., and S.C.K.: acquisition, analysis, or interpretation of data; J.K., D.W.H., and S.C.K.: drafting of the manuscript; C.M.K., J.-Y.J., Y.-S.Y., H.J.K., I.S.C., H.J.K., S.H.S., S.H.K., H.H.M., D.W.H., and S.C.K.: critical revision of the manuscript for important intellectual content; J.K.: statistical analysis; C.M.K., Y.-S.Y., and D.W.H.: administrative, technical, or material support; C.M.K., J.-Y.J., Y.-S.Y., H.J.K., I.S.C., H.J.K., S.H.S., S.H.K., H.H.M., D.W.H., and S.C.K.: supervision.

## Conflicts of interest disclosure

The authors of this work have nothing to disclose.

## Research registration unique identifying number (UIN)

Name of the registry: clinicaltrials.gov

Unique identifying number or registration ID: NCT05975710.

## Guarantor

Jaewoo Kwon and Song Cheol Kim.

## Data availability statement

Song Cheol Kim, corresponding authors, had full access to all the data in the study and takes responsibility for the integrity of the data and the accuracy of the data analysis.

## Provenance and peer review

Not commissioned, externally peer-reviewed.

## Supplementary Material

SUPPLEMENTARY MATERIAL
