# Supplementary material for: Perioperative textbook outcomes of minimally invasive pancreatoduodenectomy: a multicenter retrospective cohort analysis in a Korean minimally invasive pancreatic surgery registry
Source: Int J Surg. 2024 Apr 3;110(7):4249–58. doi: 10.1097/JS9.0000000000001390 (PMC11254279; doi:10.1097/JS9.0000000000001390)
Supplement: SUPPLEMENTARY MATERIAL [file js9-110-4249-s002.docx]

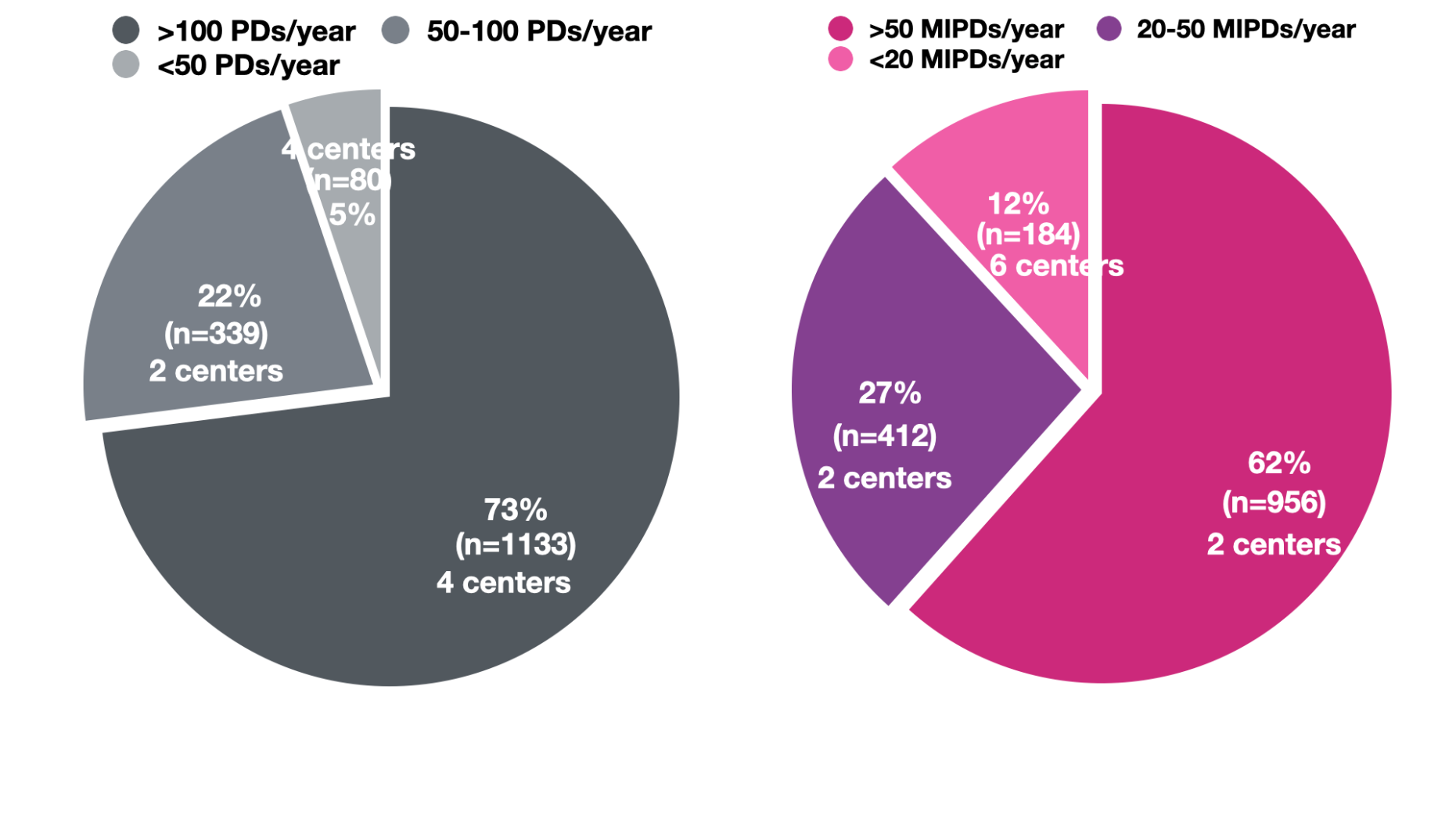


**SDC Figure 1. Percentage of Enrolled Patients by Annual Number of Surgeries in Each Institution.** An institution conducting over 50 MIPDs annually was categorized as a high-volume institution, while an institution performing fewer than 50 MIPDs was classified as a low-volume institution.


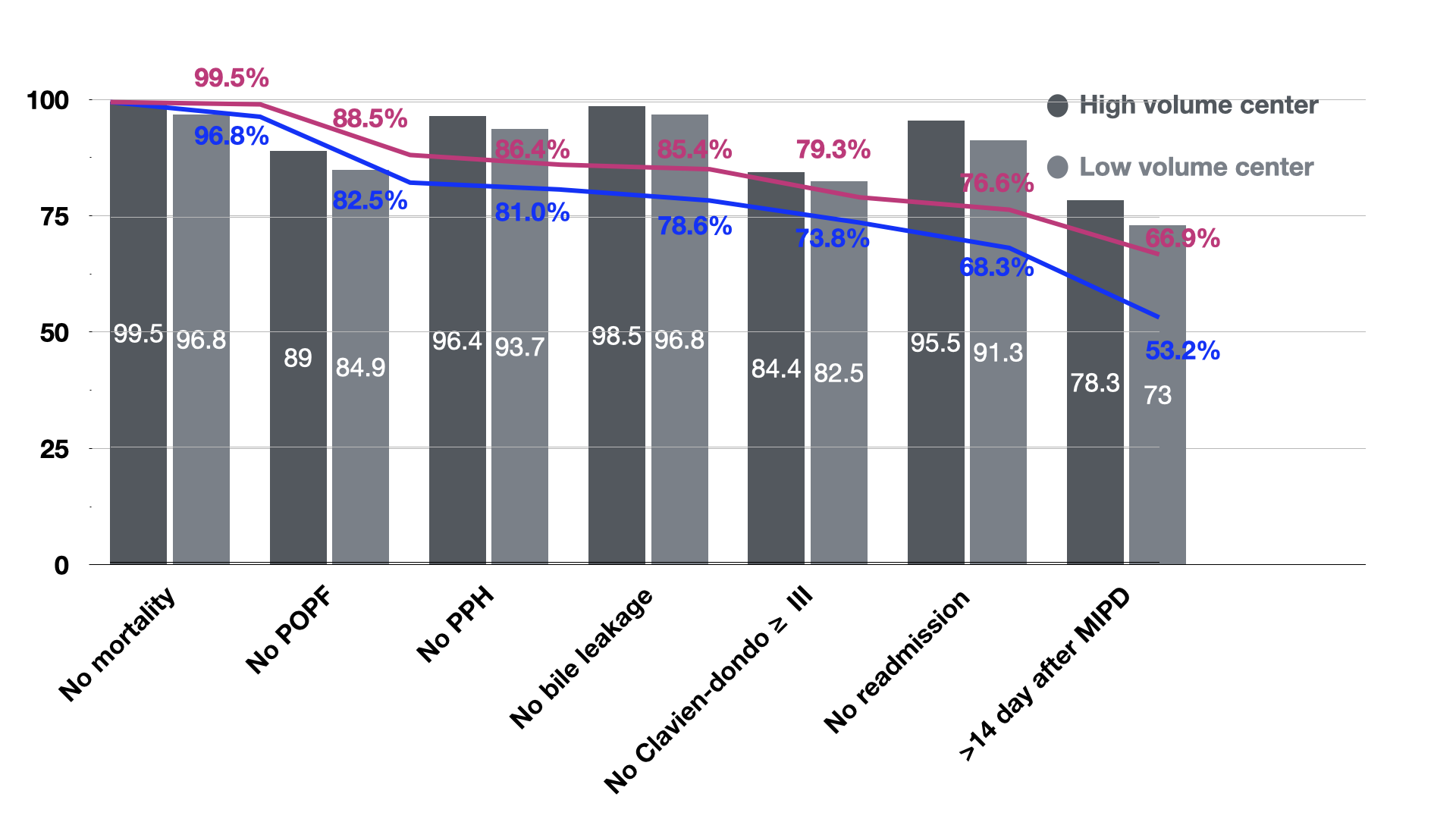


**SDC Figure 2. Textbook Outcome of Pancreatic Surgery According to Hospital Volume after excluding missing value**. The textbook outcome of pancreatic surgery is assessed individually and cumulatively. The TOPS success rate was higher in high-volume centers than in low-volume centers (66.9% vs. 53.2%, *P*=0.002).

**SDC Table 1. Study Population**

| **Variables** |  | N = 1552^a^ | Missing, N, (%) |
| --- | --- | --- | --- |
| Center, n (%) | >100 PDs/year: 4 centers  50-100 PDs/year: 2 centers  < 50 PDs/year: 4 centers | 1133 (73.0)  339 (21.8)  80 (5.2) |  |
|  | >50 MIPDs/year: 2 centers  20-50 MIPDs/year: 2 centers  <20 MIPDs/year: 6 centers | 956 (61.6)  412 (26.5)  184 (11.9) |  |
| Age, years, (±SD) | Mean  Median (IQR) | 61.5 (± 13.0)  63 (54-71) |  |
| Sex, n (%) | Female  Male | 759 (48.9)  793 (51.1) |  |
| BMI, kg/m^2^, (±SD) | Mean  Median (IQR) | 23.1 (± 2.76)  22.98 (21.26-24.91) |  |
| Previous abdominal operation history, n (%) | Yes  Minimally invasive  Open  Not available  No | 269 (20.7)  53 (4.1)  130 (10.0)  86 (6.6)  1029 (79.3) | 254  (16.4) |
| ASA score, n (%) | I  II  III  IV | 231 (14.9)  1135 (73.1)  183 (11.8)  3 (0.2) |  |
| Preoperative drainage, n (%) | Yes  Bile duct  Pancreatic duct  Both  No | 362 (37.7)  206 (21.5)  81 (8.5)  75 (7.8)  597 (62.3) | 593 (38.2) |
| Pancreas texture, n (%) | Soft  Firm | 1009 (73.4)  365 (26.6) | 178  (11.5) |
| Pancreas duct size, mm, (±SD) | Mean | 3.3 (± 2.2) |  |
| Malignancy, n (%) | Malignancy  Benign | 1147 (84.6)  209 (15.4) | 196 (12.6) |
| Operation method, n (%) | PPPD  PrPD  Conventional Whipple  Total pancreatectomy | 1373 (88.9)  102 (6.6)  42 (2.7)  27 (1.7) | 8 (0.5) |
| Type of minimally invasive surgery n (%) | Laparoscopic  Laparoscopic resection & reconstruction  Laparoscopic resection & open reconstruction  NA  Robotic  Laparoscopic resection & robotic  reconstruction  Robotic resection & reconstruction  Robotic resection & open reconstruction  NA  Open conversion | 1116 (71.9)  1018 (65.6)  42 (2.7)  56 (3.6)  339 (21.8)  286 (18.4)    49 (3.2)  2 (0.1)  2 (0.1)  97 (6.3) |  |
| Additional organ resection, n (%) | Yes  No | 32 (2.1)  1520 (97.9) |  |
| Additional major vessel resection, n (%) | Yes  Vein  Artery  Both  No | 55 (3.5)  48 (3.1)  6 (0.4)  1 (0.1)  1497 (96.5) |  |
| PJ method, n (%) | Duct to mucosa  Dunking  Inverted mattress  Blumgart  Pancreatogastrostomy  Other | 921 (72.8)  254 (20.1)  85 (6.7)  2 (0.1)  1 (0.0)  2 (0.1) | 287 (18.5) |
| Stent insertion during PJ, n (%) | Yes  No | 1498 (97.9)  32 (2.1) | 22 (1.4) |
| Transfusion, n (%) | Yes  No | 161 (10.4)  1391 (89.6) |  |
| Estimated blood loss, ml, (±SD) | Mean  Median (IQR) | 297 (± 481)  180 (100-340) |  |
| Operation time, minutes, (±SD) | Mean  Median (IQR) | 417 (± 103)  406 (345-473) |  |

Abbreviations: SD, standard deviation; BMI, body mass index; ASA score, American Society of Anesthesiology score; POPF, postoperative pancreatic fistula; ICU, intensive care unit; PPPD, pylorus preserving pancreatoduodenectomy; PrPD, pylorus resection pancreatoduodenectomy.

a Data are presented as the number (percentage) of patients unless otherwise indicated.

**SDC Table 2. Reason for open conversion**

| **Variables** |  | N = 97^a^ |
| --- | --- | --- |
| Reason, n (%) | Bleeding  Adhesion  Inflammation  Difficulty of reconstruction  Tumor advancement  Insufficient overview | 19 (19.6)  14 (14.4)  12 (12.4)  11 (11.3)  8 (8.2)  8 (8.2) |
|  | Vessel resection and reconstruction  Technical reason  Bleeding, adhesion  Inflammation, adhesion  Adhesion, vessel reconstruction  Others | 7 (7.2)  4 (4.1)  1 (1.0)  1 (1.0)  1 (1.0)  11 (11.3) |

a Data are presented as the number (percentage) of patients unless otherwise indicated.

**SDC Table 3. Demographics and Perioperative Outcomes According to MIPD cases after excluding missing value**

| **Variables** |  | Total  (n=1239) | High volume  (>20 MIPDs/year)  (n=1113) | Low volume  ($\leq$20 MIPDs/year)  (n=126) | P-value |
| --- | --- | --- | --- | --- | --- |
| Age, years, (±SD) | Mean  Median (IQR) | 61.1 (± 13.2)  62 (54-71) | 60.7 (± 13.4)  62 (53-71) | 65.2 (± 11.0)  66 (59-73) | <0.001^a^ |
| Sex, n (%) | Female  Male | 614 (49.6)  625 (50.4) | 563 (50.6)  550 (49.4) | 51 (40.5)  75 (59.5) | 0.031^b^ |
| BMI, kg/m^2^, (±SD) | Mean  Median (IQR) | 23.1 (± 2.3)  23.0 (21.3-24.9) | 23.1 (± 2.7)  23.0 (21.3-24.9) | 22.9 (± 2.8)  22.6 (21.2-24.7) | 0.413 ^a^ |
| ASA score, n (%) | I  II  III  IV | 200 (16.1)  963 (77.7)  75 (6.1)  1 (0.1) | 181 (16.3)  860 (77.3)  71 (6.4)  1 (0.1) | 19 (15.1)  103 (81.7)  4 (3.2)  0 (0.0) | 0.343^b^ |
| Operation year, n (%) | ≤2016  2017  2018  ≥2019 | 457 (36.9)  242 (19.5)  227 (18.3)  313 (25.3) | 439 (39.4)  224 (20.1)  198 (17.8)  252 (22.6) | 18 (14.3)  18 (14.3)  29 (23.0)  61 (48.4) | <0.001^b^ |
| Type of minimally invasive surgery n (%) | Laparoscopic  Robotic  Open conversion | 877 (70.8)  309 (24.9)  53 (4.3) | 769 (69.1)  301 (27.0)  43 (3.9) | 108 (80.7)  8 (6.3)  10 (7.9) | <0.001^b^ |
| Operation method, n (%) | PPPD  PrPD  Other | 1102 (88.9)  100 (8.1)  37 (3.0) | 983 (88.3)  98 (8.8)  32 (2.9) | 119 (94.4)  2 (1.6)  5 (4.0) | 0.005^b^ |
| Additional organ resection, n (%) | Yes  No | 25 (2.0)  1214 (98.0) | 21 (1.9)  1092 (98.1) | 4 (3.2)  122 (96.8) | 0.311^b^ |
| Additional major vessel resection, n (%) | Yes  No | 34 (2.7)  1205 (97.3) | 33 (3.0)  1080 (97.0) | 1 (0.8)  125 (99.2) | 0.246^b^ |
| Transfusion, n (%) | Yes  No | 131(10.6)  1108 (89.4) | 114 (10.2)  999 (89.8) | 17 (13.5)  109 (86.5) | 0.261^b^ |
| Estimated blood loss, ml, (±SD) | Mean  Median (IQR) | 281 (± 517)  100 (100-300) | 282 (± 529)  100 (100-300) | 256 (± 232)  200 (100-400) | 0.720^a^ |
| Operation time, minutes, (±SD) | Mean  Median (IQR) | 399 (± 98)  390 (332-450) | 392 (± 92)  385 (329-440) | 464 (± 121)  454 (385-517) | <0.001^a^ |
| Hospital stay after operation, days, (±SD) | Mean  Median (IQR) | 12.7 (± 10.4)  10 (8-13) | 12.6 (± 10.4)  10 (8-13) | 14.1 (± 9.9)  12 (9-15) | 0.155^a^ |
| Complications grade^+^, n (%) | None  Grade I - II  $\geq$ Grade IIIa | 556 (44.9)  487 (39.3)  196 (15.8) | 498 (44.7)  441 (39.6)  174 (15.6) | 58 (46.0)  46 (36.5)  22 (17.5) | 0.739^b^ |
| POPF, n (%) | None or biochemical leakage  Grade B or C | 1098 (88.6)  141 (11.4) | 991 (89.0)  122 (11.0) | 107 (84.9)  19 (15.1) | 0.168^b^ |
| Delayed gastric emptying, n (%) | Yes  No | 42 (3.3)  1197 (96.6) | 35 (3.1)  1078 (96.9) | 7 (5.6)  119 (94.4) | 0.156^b^ |
| Post pancreatectomy hemorrhage, n (%) | Yes  No | 48 (3.9)  1191 (96.1) | 40 (3.6)  1073 (96.4) | 8 (6.3)  118 (93.7) | 0.129^b^ |
| Chyle leakage, n (%) | Yes  No | 75 (6.1)  1164 (93.9) | 74 (6.6)  1039 (93.4) | 1 (0.8)  125 (99.2) | 0.005^b^ |
| Bile leakage, n (%) | Yes  No | 21 (1.7)  1218 (98.3) | 17 (1.5)  1096 (98.5) | 4 (3.2)  122 (96.8) | 0.260^b^ |
| Unplanned ICU care, n (%) | Yes  No | 31 (2.5)  1208 (97.5) | 20 (1.8)  1093 (98.2) | 11 (8.7)  115 (91.3) | <0.001^b^ |
| Unplanned reoperation, n (%) | Yes  No | 60 (4.8)  1179 (95.2) | 52 (4.7)  1061 (95.3) | 8 (6.3)  118 (93.7) | 0.406^b^ |
| 90-day mortality, n (%) | Yes  No | 10 (0.8)  1 (99.5) | 6 (0.5)  1107 (99.5) | 4 (3.2)  122 (96.8) | 0.013^b^ |
| 90-day readmission, n (%) | Yes  No | 61 (4.9)  1178 (95.1) | 50 (4.5)  1063 (95.5) | 11 (8.7)  115 (91.3) | 0.143^b^ |
| Textbook outcome of pancreatic surgery, n (%) | Yes  No | 812 (65.5)  427 (34.5) | 745 (66.9)  368 (33.1) | 67 (53.2)  59 (46.8) | 0.002^b^ |

Abbreviations: SD, standard deviation; BMI, body mass index; ASA score, American Society of Anesthesiology score; POPF, postoperative pancreatic fistula; ICU, intensive care unit; PPPD, pylorus preserving pancreatoduodenectomy; PrPD, pylorus resection pancreatoduodenectomy; NA, not available

^a^ P value = analysis of variance.

^b^ P value = Pearson χ2 test.

**SDC Table 4. Peri-operative Outcomes According to Number of MIPD** (>50 MIPDs/year *versus* 20-50 MIPDs/year *versus* <20 MIPDs/year).

| **Variables** |  | High volume  (>50 MIPDs /year, n=956) | Medium volume  (20-50 MIPDs/ year, n=412) | Low volume  (<20 MIPDs/ year, n=184) | P-value | Post-hoc analysis |
| --- | --- | --- | --- | --- | --- | --- |
| Age, years, (±SD) | Mean | 59.4 (± 13.1) | 64.6 (± 12.7) | 65.3 (± 11.2) | <0.001^a^ | H-M, H-L |
| Sex, n (%) | Female  Male | 486 (50.8)  470 (49.2) | 203 (49.3)  209 (50.7) | 70 (38.0)  114 (62.0) | 0.0006^b^ | H-L, M-L |
| BMI, kg/m^2^, (±SD) | Mean | 23.0 (± 2.7) | 24.0 (± 2.8) | 23.1 (± 2.9) | <0.001^a^ | H-M |
| ASA score, n (%) | I  II  III  IV | 135 (14.1)  764 (79.9)  56 (5.9)  1 (0.1) | 71 (17.2)  221(53.6)  118 (28.6)  2 (0.5) | 25 (13.6)  105 (81.5)  9 (4.9)  0 (0.0) | <0.001^b^ | H-M, H-L, M-L |
| Type of minimally invasive surgery n (%) | Laparoscopic  Robotic  Open conversion | 631 (66.0)  302 (31.6)  23 (2.4) | 332 (80.6)  29 (7.0)  51 (12.4) | 153 (83.2)  8 (4.3)  23 (12.5) | <0.001^b^ | H-M, H-L |
| Operation method, n (%) | PPPD  PrPD  Other  NA | 869 (90.9)  75 (7.8)  12 (1.2)  0 | 341 (82.8)  23 (5.6)  48 (11.7)  0 (0.0) | 163 (88.6)  4 (2.2)  9 (4.9)  8 (4.3) | <0.001^b^ | H-L, M-L |
| Additional organ resection, n (%) | Yes  No | 14 (1.5)  942 (98.1) | 13 (3.2)  399 (96.8) | 5 (2.7)  179 (97.3) | 0.104^b^ |  |
| Additional major vessel resection, n (%) | Yes  No | 29 (3.0)  927 (97.0) | 25 (6.1)  387 (93.9) | 1 (0.5)  183 (99.5) | 0.001^b^ | H-M, M-L |
| Transfusion, n (%) | Yes  No | 104 (10.9)  852 (89.1) | 20 (4.9)  392 (95.1) | 37 (20.1)  147 (79.9) | <0.001^b^ | H-M, H-L, M-L |
| Estimated blood loss, ml, (±SD) | Mean | 270 (± 568) | 335 (± 298) | 351 (± 310) | 0.044^a^ | H-M, H-L |
| Operation time, minutes, (±SD) | Mean | 386 (± 95) | 455 (± 85) | 483 (± 117) | <0.001^a^ | H-M, H-L, M-L |
| Hospital stay after operation, days, (±SD) | Mean | 12.3 (± 9.3) | 16.3 (± 13.8) | 15.2 (± 10.2) | <0.001^a^ | H-M, H-L |
| Complications grade^+^, n (%) | None  Grade I - II  $\geq$ Grade IIIa | 490 (51.3)  326 (34.1)  140 (14.6) | 125 (30.3)  217 (52.7)  70 (17.0) | 104 (56.5)  60 (32.6)  24 (13.0) | <0.001^b^ | H-M, H-L,  M-L |
| POPF, n (%) | None or biochemical  leakage  Grade B or C  NA | 850 (89.0)  105 (11.0)  1 | 362 (88.5)  47 (11.5)  3 | 115 (85.2)  20 (14.8)  49 | <0.001^b^ | H-L,  M-L |
| Delayed gastric emptying, n (%) | Yes  No  NA | 25 (2.6)  931 (97.4)  0 | 44 (10.8)  365 (89.2)  3 | 7 (5.2)  128 (94.8)  49 | <0.001^b^ | H-M, H-L,  M-L |
| Post pancreatectomy hemorrhage, n (%) | Yes  No | 36 (3.8)  920 (96.2) | 12 (2.9)  400 (97.1) | 14 (7.6)  170 (92.4) | <0.001^b^ | H-L,  M-L |
| Chyle leakage, n (%) | Yes  No  NA | 73 (7.6)  883 (92.4)  0 | 8 (2.0)  398 (98.0)  6 | 1 (0.7)  134 (99.3)  49 | <0.001^b^ | H-M, H-L |
| Bile leakage, n (%) | Yes  No | 15 (1.6)  941 (98.4) | 15 (3.6)  397 (96.2) | 4(2.2)  180 (97.8) | 0.048^b^ | H-M |
| Unplanned ICU care, n (%) | Yes  No  NA | 16 (1.7)  940 (98.3)  0 | 4 (2.5)  154 (97.5)  254 | 12 (8.9)  123 (91.1)  49 | <0.001^b^ | H-L,  M-L |
| Unplanned reoperation, n (%) | Yes  No  NA | 44 (4.6)  912 (95.4)  0 | 8 (5.1)  150 (94.9)  254 | 9 (6.7)  126 (93.3)  49 | 0.126^b^ |  |
| 90-day mortality, n (%) | Yes  No  NA | 5 (0.5)  951 (99.5)  0 | 2 (0.5)  410 (99.5)  0 | 5 (2.7)  178 (97.3)  1 | <0.001^b^ | H-L,  M-L |
| 90-day readmission, n (%) | Yes  No  NA | 33 (3.5)  922 (96.5)  1 | 28 (6.8)  383 (93.2)  1 | 15 (9.6)  142 (90.4)  27 | <0.001^b^ | H-M, H-L,  M-L |
| Textbook outcome of pancreatic surgery, n (%) | Yes  No  NA | 651 (68.2)  304 (31.8)  1 | 200 (48.7)  211 (51.3)  1 | 69 (43.9)  88 (56.1)  27 | <0.001^b^ | H-M, H-L |

Abbreviations: SD, standard deviation; BMI, body mass index; ASA score, American Society of Anesthesiology score; POPF, postoperative pancreatic fistula; ICU, intensive care unit; PPPD, pylorus preserving pancreatoduodenectomy; PrPD, pylorus resection pancreatoduodenectomy; NA, not available; H, High volume center; M, Medium volume center; L, Low volume center

^a^ P value = ANOVA test.

^b^ P value = Pearson χ2 test.
